# Changes in inflammatory cytokine networks in myasthenia gravis

**DOI:** 10.1038/srep25886

**Published:** 2016-05-13

**Authors:** Akiyuki Uzawa, Tetsuya Kanai, Naoki Kawaguchi, Fumiko Oda, Keiichi Himuro, Satoshi Kuwabara

**Affiliations:** 1 Department of Neurology, Graduate School of Medicine, Chiba University, Japan; 2 Department of Neurology, Neurology Chiba Clinic, Japan

## Abstract

Myasthenia gravis (MG) is an autoimmunological inflammatory disorder of the neuromuscular junction. Inflammation could be a key player for understanding the pathogenesis of MG. We measured the serum levels of 24 inflammatory cytokines in 43 patients with anti-acetylcholine receptor antibody-positive MG and 25 healthy controls. In patients with MG, serum levels of a proliferation-inducing ligand (APRIL), IL-19, IL-20, IL-28A and IL-35 were significantly increased as compared with controls (p < 0.05). Among them, IL-20, IL-28A and IL-35 were significantly decreased after treatment (p < 0.05). In clinical subtype analyses, APRIL and IL-20 were increased in patients with late-onset MG and IL-28A levels were increased in patients with thymoma-associated MG compared with healthy controls (p < 0.01). The results of the present study demonstrate both anti-inflammatory and inflammatory cytokines are upregulated in MG, reflecting the importance of cytokine-mediated inflammation and its regulation in MG pathophysiology.

Myasthenia gravis (MG) is an autoimmune-mediated disease of the neuromuscular junction and is clinically characterised by muscle weakness with fatigability^1^. The majority of patients with MG have autoantibodies against muscle nicotinic acetylcholine receptor (AChR), which is produced by T cell-dependent and B cell-mediated mechanisms^1^, and triggers inflammation via complement activation at the postsynaptic muscle membrane^2^. Growing evidence indicates the presence of inflammation and the modulation of immune response by cytokines contribute to MG pathogenesis^3^,^4^,^5^. A previous study reported serum interleukin (IL)-17 levels are significantly increased and associated with MG severity^6^,^7^ and that serum IL-22 levels are decreased and negatively correlated with anti-AChR antibody levels in patients with MG^7^. We have previously demonstrated significantly increased IL-15 and VEGF levels and significantly decreased IL-4 levels in patients with MG, in addition to a significant increase in IL-8, eotaxin, MIP-1α, MIP-1β and IL-1b levels in MG patients with thymoma^3^. Although the importance of cytokines in MG pathogenesis has been established, the underlying mechanisms are yet to be fully elucidated. The present study aimed to analyze serum levels of representative inflammation-related cytokines in patients with anti-AChR antibody-positive MG.

## Materials and Methods

### Subjects

Forty-three Japanese patients with anti-AChR antibody positive MG and 25 healthy participants of similar age and sex (men, 9; women, 16; mean age, 52.4 years; range, 33–72 years) were included in the present study. We reviewed patient data regarding sex, age, disease duration, anti-AChR antibody titer, E-L-T classification (early-onset MG [EOMG], age at onset ≤49 years; late-onset MG [LOMG], age at onset ≥50 years; thymoma-associated MG [TAMG])^8^, MG Foundation of America (MGFA) classification^9^ and quantitative MG (QMG) scores^9^ at the time of serum sampling. No patients with MG received immunosuppressive therapy then. Serum samples were obtained from 10 patients with MG after initiation of immunosuppressive therapy.

Ethical approval was granted by the Ethics Committee of the Chiba University School of Medicine, Chiba, Japan and all experiments were performed in accordance with relevant guidelines and regulations. All subjects provided written informed consent for their participation in the present study.

### Serum cytokine analysis

All serum samples were stored at −80 °C until analysis. Samples were simultaneously analyzed for the following cytokines: APRIL (a proliferation-inducing ligand)/TNFSF13; B-cell activating factor of the tumor necrosis factor family (BAFF)/TNFSF13B; sCD30/TNFRSF8; sCD163; Chitinase-3-like 1; gp130/sIL-6Rβ; IFN-β; IL-11; IL-19; IL-20; IL-26; IL-27 (p28); IL-28A/IFN-λ2; IL-29/IFN-λ1; IL-32; IL-34; IL-35; LIGHT/TNFSF14; Osteocalcin; Pentraxin-3; sTNF-R1; sTNF-R2; TSLP and TWEAK/TNFSF12 using a 24-plex Bio-Plex Pro™ Human Inflammation Panel 1 (Bio-Rad Laboratories, Inc., Hercules, CA, USA) according to the manufacturer’s instructions. Values were recorded as the lower or upper detection limits in cases where serum values were outside the detection range.

### Statistical analysis

The groups were compared using the Mann–Whitney *U* test for unpaired continuous measures and the Wilcoxon signed-rank test for paired continuous measures. For multiple comparisons, Bonferroni’s correction was applied. The Spearman’s rank correlation coefficient was used to test associations between datasets.

## Results

### Clinical profiles of patients with MG

The clinical characteristics of patients with MG at the time of serum sampling were as follows: female ratio, 26/43 (60.5%); mean age ± SD (range), 56.0 ± 17.0 (23–79) years; mean disease duration, 12.8 ± 18.7 (0–81) months; mean anti-AChR antibody titer, 98.8 ± 191.6 (0.3–1100) nmol/L; E-L-T classification, EOMG = 14, LOMG = 18, TAMG = 11; median MGFA classification, 2 (1–5); mean QMG score, 9.7 ± 5.8 (1–25) points.

### Serum cytokines levels in patients with MG

**Figure 1:**
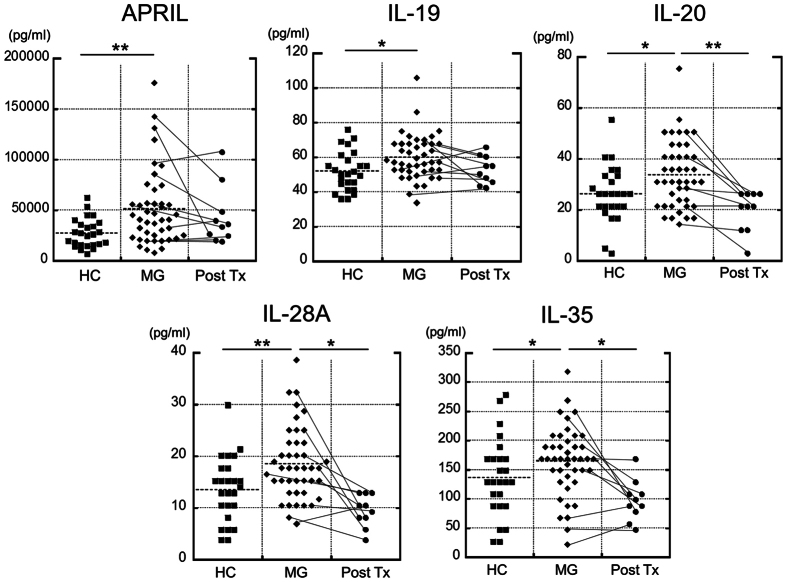
Serum cytokine levels in patients with anti-AChR antibody-positive myasthenia gravis (MG) and healthy controls (HC). Serum levels of APRIL, IL-19, IL-20, IL-28A and IL-35 were significantly elevated in patients with MG compared to HC (Mann–Whitney *U* test, p < 0.05). Serum levels of IL-20, IL-28A and IL-35 were significantly decreased following treatment (Post-Tx; Wilcoxon signed-rank test; p < 0.05). Dashed lines indicate mean serum levels in each group. **p < 0.01, *p < 0.05.

**Figure 2:**
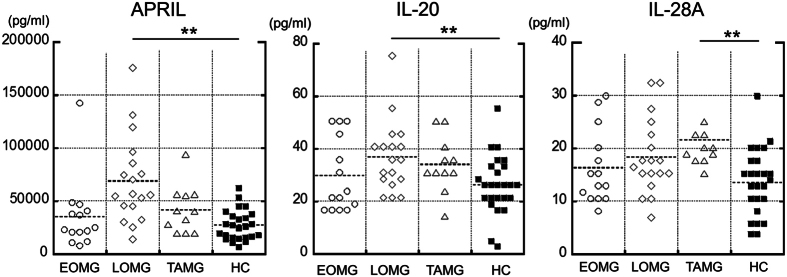
Serum cytokine levels in early-onset myasthenia gravis (EOMG), late-onset MG (LOMG), thymoma-associated MG (TAMG) and healthy controls (HC). Serum levels of APRIL and IL-20 were elevated in LOMG. Serum levels of IL-28A were elevated in TAMG compared to HC (p < 0.01). Dashed lines indicate mean serum levels in each group. **p < 0.01.

Of the 24 measured cytokines, serum levels of APRIL (p = 0.002); IL-19 (p = 0.013); IL-20 (p = 0.031); IL-28A (p = 0.008) and IL-35 (p = 0.042) were significantly higher in all patients with MG compared to healthy controls (HC) (Fig. 1 and Table 1). Of the significantly changed cytokines (APRIL, IL-19, IL-20, IL-28A and IL-35), only IL-20 (p = 0.008), IL-28A (p = 0.013) and IL-35 (p = 0.036) levels were significantly decreased after immunosuppressive therapy in 10 patients with MG (Fig. 1). When MG patients were categorised according to E-L-T classification, serum levels of APRIL (p < 0.001) and IL-20 (p = 0.006) were significantly higher in patients with LOMG than in HC and levels of IL-28A (p < 0.001) were significantly higher in patients with TAMG than in HC even after Bonferroni’s correction (Table 1 and Fig. 2).

Significant correlations were observed between serum levels of APRIL, IL-19, IL-20, IL-28A and IL-35 (Table 2). No significant correlation was observed between clinical manifestations of MG and the serum levels of the above-described cytokines (data not shown).

## Discussion

The results of the present study demonstrated significant increases in the serum levels of APRIL, IL-19, IL-20, IL-28A and IL-35 in patients with anti-AChR antibody-positive MG compared to HC. Moreover, serum levels of IL-20, IL-28A and IL-35 were significantly decreased after immunosuppressive therapy.

Cytokines, in addition to anti-AChR antibodies and complement proteins, play a major role in the development of inflammation at the neuromuscular junction in MG. A previous study in an experimental autoimmune myasthenia gravis model demonstrated that increased proportion of Th1 (type 1 helper T cell) and Th17 cells worsen MG pathogenesis, whereas increased proportions of Th2 and Treg (regulatory T cell) cells ameliorate MG development^10^. Similar results have been reported in studies of patients with MG, including the upregulation of IL-17 levels^6^ and downregulation of IL-4 levels^3^.

IL-10, IL-22, IL-24, IL-26, IL-28 and IL-29 are classified as cytokines belonging to the IL-10 family, which play critical roles in inflammatory diseases^11^. Members of the IL-10 family share a number of common features, such as similar genomic/protein structures and receptor complexes. Of these, we confirmed that levels of IL-19, IL-20 and IL-28A were significantly increased in MG. To date, there have been no studies examining the levels of these cytokines in MG. IL-19 and IL-20, members of the IL-20 subfamily of IL-10 family, may be involved in development of inflammation in MG. IL-19 has been shown to activate immune cells, stimulate the release of pro-inflammatory cytokines (e.g., IL-6 and TNF-α)^12^ and upregulate Th2 responses^13^. Conversely, IL-19 knockout mice have recently been reported to develop exacerbations of colitis^14^ and IL-19 deficiency has been shown to increase the production of pro-inflammatory cytokines in activated microglia^15^, indicating the immunopathological relevance of IL-19 as an anti-inflammatory cytokine. IL-20 acts as a proinflammatory cytokine and has been shown to be upregulated in the synovial fluid of patients with rheumatoid arthritis^16^ and in the psoriatic lesions^17^ and to be associated with lupus nephritis^18^. In addition, IL-19 and IL-20 enhance tissue remodeling activities and angiogenesis in response to inflammation^19^. VEGF, which is an important cytokine in the promotion of angiogenesis, has also been shown to be upregulated in MG^3^. Elevations in the serum levels of these cytokines in MG may reflect the presence of angiogenesis in response to inflammation. Further studies are required to validate the functions of these cytokines in MG pathogenesis.

IL-28A is known as a type III interferon and has potent antiviral effects. IL-28 promoted Th1 skewing and inhibited Th2 and Th17 responses in an allergic asthma model^20^ and IL-28-treated dendritic cells induced the proliferation of Treg cells^21^. Therefore, elevated IL-28 levels may have a protective role in MG pathogenesis. Regarding other IL-10 family cytokines, IL-10, which is a representative anti-inflammatory cytokine and IL-22 are reportedly downregulated in patients with MG. In patients with MG, B cell-derived IL-10 is downregulated^22^, whereas serum IL-10 levels tended to be upregulated^3^. Serum IL-22 levels were downregulated and negatively correlated with anti-AChR antibody titers in patients with MG^7^. These results indicate IL-10 family cytokines have multivalent functions and may play essential roles in mechanisms underlying the development of inflammation in MG.

IL-35, which is a member of the IL-12 family, is produced by the Treg cells and functions as an anti-inflammatory cytokine^23^. Although no previous studies have demonstrated an association between IL-35 and the development of MG, the results of the present study indicate IL-35 may perform anti-inflammatory and protective functions in MG.

APRIL, which is a member of the TNF-ligand superfamily, is secreted by macrophages, T cells and dendritic cells and has an important role in the maturation and survival of B cells^24^. B cells are responsible for the production of autoantibodies against AChR in MG. A previous study reported no difference in APRIL and BAFF levels between patients with MG and controls^25^. Thymic expression of APRIL and BAFF may represent a source of these cytokines in MG. Interestingly, APRIL-positive cells have been confirmed in hyperplastic thymus and atrophic thymus but were scant in thymoma^25^, which may explain the difference in APRIL levels between EOMG, LOMG and TAMG. The results indicate that the upregulation of APRIL in MG may influence the production of autoantibodies by B cells.

The present study had some limitations. The sample size of patients with MG, especially in the MG subgroups, was relatively small, which may limit the generalisability of the present study. We were unable to demonstrate significant correlations between serum cytokines levels and clinical parameters. Further, serum cytokines levels may not necessarily reflect those levels at neuromuscular junction. Further studies are required to address these limitations.

In conclusion, the results of the present study demonstrate significantly increased serum levels of pro- and anti-inflammatory cytokines in patients with MG. Several inflammation-related cytokines may form complicate networks and play an important role in mediating pathogenic and inflammatory mechanisms at the neuromuscular junction in the pathogenesis of MG. Greater understanding of cytokine signaling pathways may facilitate the development of novel treatments for MG.

## Additional Information

**How to cite this article**: Uzawa, A. *et al*. Changes in inflammatory cytokine networks in myasthenia gravis. *Sci. Rep.* **6**, 25886; doi: 10.1038/srep25886 (2016).

## Figures and Tables

**Table 1:** Serum cytokines levels in patients with MG.

Cytokines, pg/mL	All MG (n = 43)	Early onset MG (n = 14)	Late onset MG (n = 18)	Thymoma-associated MG (n = 11)	Healthy control (n = 25)
APRIL	**50973.5** **±** 37302.2*	35181.2 ± 33519.2	**68875.0** **±** **40996.6**†	41779.4 ± 22523.1	27222.3 ± 14240.6
BAFF	12368.2 ± 4433.1	11307.8 ± 4398.8	12677.9 ± 3934.6	13210.9 ± 5342.2	10913.6 ± 2597.7
sCD30	727.7 ± 564.0	638.9 ± 310.3	644.7 ± 344.1	976.7 ± 958.1	538.6 ± 206.4
sCD163	140026.5 ± 56441.1	128514.6 ± 65562.9	146872.4 ± 51440.8	143475.6 ± 54863.5	120921.6 ± 46039.2
Chitinase-3-like 1	22209.2 ± 16911.9	17180.6 ± 13530.2	27745.9 ± 20122.9	19549.1 ± 13286.0	19753.6 ± 15950.7
gp130	87615.8 ± 32470.2	85774.6 ± 37294.1	91303.4 ± 35859.0	83925.0 ± 19744.3	93750.6 ± 33019.6
IFN-β	19.7 ± 14.2	15.9 ± 14.3	21.9 ± 14.5	21.1 ± 13.8	14.5 ± 10.3
IL-11	0.4 ± 0.4	0.3 ± 0.0	0.5 ± 0.5	0.4 ± 0.5	0.4 ± 0.5
IL-19	**59.9** **±** 12.9*	58.4 ± 8.7	60.7 ± 16.2	60.4 ± 12.3	52.0 ± 11.9
IL-20	**33.9** **±** 13.1*	29.7 ± 14.1	**37.0** **±** **13.5**†	34.1 ± 10.6	26.3 ± 11.1
IL-26	9.3 ± 0.8	9.2 ± 0.0	9.5 ± 1.2	9.2 ± 0.0	9.2 ± 0.0
IL-27 (p28)	14.7 ± 25.6	10.4 ± 0.0	20.7 ± 39.4	10.4 ± 0.0	10.7 ± 1.4
IL-28A	**18.5** **±** 7.0*	16.3 ± 7.0	18.4 ± 7.1	**21.5** **±** **6.3**†	13.6 ± 6.2
IL-29	15.2 ± 9.9	13.3 ± 10.4	15.5 ± 11.2	17.0 ± 7.2	13.0 ± 10.4
IL-32	12.3 ± 27.3	4.7 ± 4.4	21.6 ± 40.1	6.7 ± 10.0	17.3 ± 27.0
IL-34	63.3 ± 44.4	54.3 ± 0.0	75.8 ± 67.8	54.3 ± 0.0	55.1 ± 3.7
IL-35	**165.1** **±** 58.8*	144.2 ± 61.1	175.6 ± 48.7	174.6 ± 68.9	136.3 ± 67.1
LIGHT	46.6 ± 70.7	40.3 ± 54.4	56.5 ± 94.0	38.4 ± 42.7	46.7 ± 65.5
Osteocalcin	3733.5 ± 2173.3	4455.1 ± 2719.3	3404.1 ± 2076.0	3353.9 ± 1347.6	4199.5 ± 1395.8
Pentraxin-3	1532.0 ± 1429.4	1272.0 ± 502.4	1864.8 ± 2099.2	1318.5 ± 646.7	1167.8 ± 752.9
sTNF-R1	5696.3 ± 2496.2	5310.7 ± 2031.5	6441.7 ± 2964.8	4967.2 ± 2017.1	4870.8 ± 2267.6
sTNF-R2	6882.5 ± 4320.3	6064.8 ± 3751.6	7423.5 ± 4699.4	7037.7 ± 4641.2	5449.3 ± 3786.4
TSLP	13.9 ± 10.3	10.8 ± 9.4	16.8 ± 10.1	13.3 ± 11.2	9.5 ± 6.2
TWEAK	728.7 ± 145.6	741.6 ± 120.4	686.6 ± 149.1	781.3 ± 161.0	708.4 ± 201.3

APRIL, a proliferation-inducing ligand; BAFF, B-cell activating factor of the tumor necrosis factor family. IL, interleukin; Values are presented as means ± standard deviation.

^*^Significant difference compared to HC (P < 0.05).

^†^Significant difference compared to HC after Bonferroni’s correction (P < 0.01).

**Table 2:** Correlations among cytokines.

	IL-19	IL-20	IL-28A	IL-35
APRIL	r = 0.275	r = 0.498	r = 0.427	r = 0.522
	(p = 0.075)	(p = 0.001*)	(p = 0.006*)	(p = 0.001*)
IL-19	–	r = 0.387	r = 0.247	r = 0.342
		(p = 0.012*)	(p = 0.110)	(p = 0.027*)
IL-20	–	–	r = 0.771	r = 0.667
			(p < 0.001*)	(p < 0.001*)
IL-28A	–	–	–	r = 0.676
				(p < 0.001*)

IL, interleukin.

^*^Significant difference according to Spearman’s rank correlation coefficient.
